# Intravoxel incoherent motion diffusion-weighted imaging for discriminating the pathological response to neoadjuvant chemoradiotherapy in locally advanced rectal cancer

**DOI:** 10.1038/s41598-017-09227-9

**Published:** 2017-08-17

**Authors:** Wen Lu, Hou Jing, Zhou Ju-Mei, Nie Shao-Lin, Cao Fang, Yu Xiao-Ping, Lu Qiang, Zeng Biao, Zhu Su-Yu, Hu Ying

**Affiliations:** 1 Department of Diagnostic Radiology, Hunan Cancer Hospital and the Affiliated Cancer Hospital of Xiangya School of Medicine,Central South University, Changsha, 410013 Hunan P.R. China; 2 Department of Radiotherapy, Hunan Cancer Hospital and the Affiliated Cancer Hospital of Xiangya School of Medicine,Central South University, Changsha, 410013 Hunan P.R. China; 3 Department of Colorectal Surgery, Hunan Cancer Hospital and the Affiliated Cancer Hospital of Xiangya School of Medicine,Central South University, Changsha, 410013 Hunan P.R. China; 4 Department of Pathology, Hunan Cancer Hospital and the Affiliated Cancer Hospital of Xiangya School of Medicine, Central South University, Changsha, 410013 Hunan P.R. China

## Abstract

To investigate the usefulness of intravoxel incoherent motion diffusion-weighted imaging (IVIM-DWI) in discriminating the pathological complete response (pCR) to neoadjuvant chemoradiotherapy (nCRT) in locally advanced rectal cancer (LARC), 42 patients underwent preoperative IVIM-DWI before (pre-nCRT) and after nCRT (post-nCRT). The values of pre-nCRT and post-nCRT IVIM-DWI parameters (ADC, D, D* and *f*), together with the percentage changes (∆% parametric value) induced by nCRT, were compared between the pCR (tumour regression grade [TRG] 4) and non-pCR (TRG 0, 1, 2 or 3) groups and between the GR (TRG 3 or 4) and PR (TRG 0, 1 or 2) groups based on the Dworak TRG system. After nCRT, the ADC and D values for LARC increased significantly (all *P* < 0.05). The TRG score revealed a positive correlation with pre*f* (r = 0.357, *P* = 0.020), postD (r = 0.551, *P* < 0.001) and Δ%D (r = 0.605, *P* < 0.001). The pCR group (n = 10) had higher preD*, pre*f*, postD, ∆%ADC and ∆%D values than the non-pCR group (n = 32) (all *P* < 0.05). The GR group (n = 15) exhibited higher postD, ∆%ADC and ∆%D values than the PR group (n = 27) (all *P* < 0.05). Based on ROC analysis, ∆%D had a higher area under the curve value than ∆%ADC (*P* = 0.009) in discriminating the pCR from non-pCR groups. In conclusion, IVIM-DWI may be helpful in identifying the pCR to nCRT for LARC and is more accurate than traditional DWI.

## Introduction

Neoadjuvant chemoradiotherapy (nCRT) followed by total mesorectal excision (TME) has become a standard treatment in patients with locally advanced rectal cancer (LARC)^1^, which could decrease the loco-regional recurrence rate and even increase overall survival. After nCRT, approximately 15%–27% of LARC patients achieved a pathologic complete response (pCR)^2, 3^. These patients have a favourable long-term outcome with excellent local control and disease-free survival. Moreover, Maas and Intven M found that patients with a pCR have an excellent long-term outcome regardless of surgical resection^2, 3^. Therefore, some investigators have suggested that surgery can be omitted in patients achieving a pCR to nCRT. On the other hand, patients achieving a pathologic poor response (PR) may benefit less from nCRT but suffer its toxicity compared with those with a good response (GR). Thus, accurately identifying a pathologic response before or at the early stage of nCRT would have great significance for personalized treatment.

Currently, magnetic resonance imaging (MRI) has become an important approach for assessing the response to nCRT for LARC, for which MRI-based tumour volume measurement and T-downstaging analysis are widely used^2, 4^. Nevertheless, MRI volumetric evaluation and T-downstaging analysis have obvious limitations in identifying the tumour response to nCRT, owing to similar morphological appearances^4^. Moreover, changes in tumour morphology on MRI often develop later than those in microenvironmental function as a therapeutic effect due to nCRT. In recent years, diffusion-weighted imaging (DWI), with its ability to quantify the diffusion motion of water molecules, has proven to be potentially helpful in predicting the response to nCRT in LARC^5–10^. However, the potency of the apparent diffusion coefficient (ADC) derived from traditional DWI on the basis of a mono-exponential decay model was inconsistent across different studies^6, 7, 11, 12^. In fact, the motion of water molecules in viable tissues is influenced by both thermally driven motion (pure diffusion) and microcirculation blood perfusion.

Dynamic contrast-enhanced MRI (DCE-MRI) provides information regarding the microcirculation perfusion of tissues. Previous studies have described the potential of DCE-MRI for predicting the treatment response of rectal cancer^3, 13^. However, DCE-MRI requires the administration of exogenous gadolinium-containing contrast agent, which is costly and associated with some medical risks, including allergy and nephrogenic systemic fibrosis, limiting the clinical application of DCE-MRI.

Based on the bi-exponential model^14^, intravoxel incoherent motion DWI (IVIM-DWI) can separately quantitate the pure diffusion motion and perfusion- related motion of water molecules without using an exogenous contrast agent. Theoretically, IVIM-DWI may characterize the microenvironmental information of tissues more accurately than conventional DWI. Recently, several studies have demonstrated that IVIM-DWI has an advantage over conventional DWI in monitoring the treatment response for various tumours, such as nasopharyngeal carcinoma^15^ and breast cancer liver metastases^16^. However, to the best of our knowledge, the feasibility of IVIM-DWI in identifying the tumour pathological response of LARC to nCRT has not been well determined, especially discriminating pCR from non-pCR, although a recent study investigated the utility of IVIM-DWI in separating PR from GR. Therefore, the purpose of the present study was to evaluate the utility of IVIM-DWI in discriminating the pathological response to nCRT for LARC.

## Materials and Methods

### Patient Selection

This prospective single-centre study was performed in accordance with the Declaration of Helsinki. The protocol for this study was approved by the Medical Ethics Committee of our institution (IRB Protocol Number: 2015-03). Written informed consent was obtained from all patients. The inclusion criteria were (1) newly diagnosed rectal non-mucinous adenocarcinoma confirmed by endoscopic biopsy, (2) being scheduled for nCRT before surgical resection, (3) clinical stage of II to III (cT3-4M0 and/or regional lymph node positive), and (4) age more than 18 years. The exclusion criteria were (1) prior anti-tumour therapy, (2) absence of a signed informed consent form, or (3) any contraindication for MRI or nCRT. From June 2015 to September 2015, 45 LARC patients were initially enrolled into the present study. The management for all LARC patients was based on the National Comprehensive Cancer Network (NCCN) Guidelines Version 3.2014.

### Conventional MRI Protocol

All patients received conventional MRI examinations at baseline (1–3 days before nCRT) and 8 weeks after the end of nCRT (i.e., 2–5 days before surgical resection). All MRI examinations were performed using a 1.5-Tesla MRI scanner (Optima® MR 360; GE Medical System, Milwaukee, WI) using a phased-array body coil. The conventional MRI protocol included the following: (1) axial T1-weighted fast spin-echo (FSE) images [time of repetition (TR), 4694 ms; time of echo (TE): 102 ms; slice thickness: 5 mm; intersection space: 1 mm; field of view (FOV): 380 mm; acquisition matrix: 320 × 224; number of excitations (NEX): 2]; (2) axial T2-weighted FSE images [TR: 4435 ms; TE: 102 MS; slice thickness: 5 mm; intersection space: 1 mm; FOV: 380 mm; acquisition matrix: 320 × 224; NEX: 2]; (3) high-resolution T2-weighted FSE images perpendicular to the longitudinal axis of the rectal tumour [TR: 4500 ms; TE: 102 ms; slice thickness: 3 mm; intersection space: 0.5 mm; FOV: 256 mm; matrix: 200 × 200; NEX: 4]; (4) sagittal high-resolution T2-weighted FSE images [TR: 4500 ms; TE: 102 ms; slice thickness: 3 mm; intersection space: 0.5 mm; FOV: 256 mm; matrix: 200 × 200, NEX: 4].

### IVIM-DWI Protocol

IVIM-DWI was performed after the conventional MRI examinations. A total of 12 b values (0, 10, 20, 30, 50, 80,100, 150, 200, 400, 600 and 800 s/mm^2^) were applied with a single-shot diffusion-weighted spin-echo echo-planar (ssSE-DW-EPI) sequence. The lookup table of gradient direction was modified to allow multiple b value measurements in one series. Parallel imaging was used with an acceleration factor of 2. In total, 20 axial slices covering the pelvic area were obtained with a 38 × 30-cm FOV, a 3-mm slice thickness, a 0.5-mm slice gap, a 4500-ms TR, a 97-ms TE, a matrix of 128 × 130, and an NEX of 4.

### IVIM-DWI Analysis

All IVIM-DWI data were transferred to an Advantage Workstation with Functool software (version ADW 4.6; GE Medical Systems) for post-processing. IVIM-DWI analysis was performed using the MADC kit, a software package for multiple ADC measurements in the Functool software package, and was fitted on a pixel-by-pixel basis according to the Levenberg-Marquardt algorithm^17^. Briefly, the major procedures of the IVIM analysis were as follows:

According to the IVIM theory described by Le Bihan^18^, the signal intensities and b values are related as follows:

1 $${{\rm{S}}}_{{\rm{b}}}/{{\rm{S}}}_{0}=(1-f)\exp (-{\rm{bD}})+f\,\exp (-{\mathrm{bD}}^{\ast })$$

where S_b_ is the signal intensity with diffusion gradient b; S_0_ is the signal intensity for b = 0 s/mm²; D is the true diffusion coefficient indicating the pure diffusion of water molecules caused by Brownian movement; *f* is the microvascular volume fraction, representing the fraction of diffusion related to microcirculation perfusion; and D* is the pseudo-diffusion coefficient due to microcirculation perfusion. Because D* is approximately one order of magnitude greater than D^18^, −bD* would be less than −3 at a high b value (>200 s/mm²), and the term *f* exp(−bD*) would be less than 0.05 *f*. In this case, the contribution of D* to the signal ratio S_b_/S_0_ can be neglected, and Eq. (1) was simplified to Eq. (2) for the estimation of D:

2 $${{\rm{S}}}_{{\rm{b}}}/{{\rm{S}}}_{0}=(1-f)\exp (-{\rm{bD}}).$$

Thus, for IVIM-DWI data at high b values (400, 600 and 800 s/mm^2^), S_b_ was first fitted to Eq. (2) using a linear model, and D was calculated. Second, fixing D at the value estimated above and considering measurements from all b values, D* and *f* were determined from Eq. (1) using a nonlinear Levenberg-Marquardt method^17^. Finally, the ADC was calculated from the traditional ADC equation, Eq. (3), using IVIM-DWI data at b values of 0, 200, 400, 600 and 800 s/mm².

3 $${{\rm{S}}}_{{\rm{b}}}/{{\rm{S}}}_{0}=\exp (-{\rm{bADC}})$$

IVIM-DWI analysis was independently and double blindly performed by two observers (W.L. and H.J., with 10 and 8 years of experience in abdomen radiology, respectively) who were blinded to the results of the treatment response. Next, 3 regions of interest (ROIs) were manually drawn by each observer for each tumour on DWI images (b = 800 s/mm²) at its widest section plus adjacent up and down sections, avoiding visually large cystic and necrotic areas, and were then subsequently co-registered to IVIM-DWI maps for further analysis. Each IVIM-DWI metric value was acquired by each observer, and correspondingly, two initial data points were generated, each of which was the average of the values obtained from the 3 ROIs by one observer. The eventual metric value for each tumour was the mean value of the two initial data points. Additionally, the two initial data points were used to evaluate the inter-observer reproducibility.

### nCRT Treatment

Intensity-modulated radiation therapy (IMRT) was performed on all patients for 5 weeks, accompanied by concurrent chemotherapy with oral capecitabine (1,650 mg/m² body-surface area) daily. All patients were simulated on a computed tomography (CT) simulator. Two physicists (Z.J. and H.Y.) who specialized in clinical oncology and radiotherapy, together with one radiologist (Y.X.), participated in the target area delineation. The gross tumour volume (GTV) was defined using all information from clinical examination, colonoscopy, pelvic MRI and CT, plus FDG-PET, if available. The GTV covered rectal lesions and any suspicious metastatic lymph nodes. The CTV encompassed the GTV as well as the peri-rectal, pre-sacral and internal iliac lymph node regions. External iliac nodal regions were also included in the CTV for T4 tumours involving anterior structures. For low rectal tumours, the CTV also included the sciatic rectal fossa to encompass the pudendal and inferior rectal nodes. Next, the planning target volume (PTV) was defined as an additional 1.0 cm beyond the scope of the CTV to allow for internal organ motion and setup error. The radiotherapy scheme was produced by one physicist (Z.B.) per the prescribed dose and was approved by 2 radiotherapy experts in rectal cancer (Z.S. and H.Y.). The prescription dose for the PTV was 45 Gy/25 fractions (1.8 Gy/fraction, 1 fraction/day, 5 fractions/week); for the GTV, it was 50 Gy/25 fractions (2.0 Gy/fraction, 1 fraction/day, 5 fractions/week). The limitations of the organs-at-risk were as follows: bladder V50 ≤ 50%; small intestinal V20 ≤ 50%; Dmax ≤ 50 Gy; and bilateral femoral heads V50 ≤ 5%. IMRT was performed on a 6-MV X-ray linear accelerator (Elekta Synergy®, Stockholm, Sweden).

### Pathological Response Evaluation

TME was performed after post-nCRT MRI examinations. After TME, the fresh specimens were fixed in formalin for 48 hours. Tissue sections stained with haematoxylin-eosin were evaluated by one pathologist (C.F., with 10 years of experience in colorectal pathology). Postoperative tumour staging was performed according to the American Joint Committee on Cancer (AJCC) TNM system^19^. The pathologic response induced by nCRT was categorized according to the Dworak tumour regression grade (TRG) system as follows^20^: TRG 4, absence of residual cancer, only a fibrotic mass (complete response); TRG 3, presence of rare residual cancer cells scattered through the fibrosis; TRG 2, increased number of residual cancer cells, but still predominating fibrosis; TRG 1, residual cancer outgrowing fibrosis; TRG 0, absence of regression changes (no response). In this study, the patients with TRG 4 were categorized as the pathological complete responder (pCR) group, whereas the non-pathological responder (non-pCR) group consisted of those with other TRG scores. We also classified the patients into the GR (TRG 3 or 4) and PR (TRG 0, 1 or 2) groups.

### Statistical Analysis

The intra-class correlation coefficients were calculated to evaluate inter-observer variability. The percentage changes in the IVIM-DWI parametric values were calculated by dividing the mathematical difference in the corresponding parametric values before and after nCRT by the parametric value before nCRT—for example, ∆%D = (postD − preD)/preD × 100%. The differences in the IVIM-DWI parametric values before and after nCRT for all patients were explored by the Wilcoxson signed-rank test. The Mann-Whitney U test was used to investigate the differences in the IVIM-DWI parametric values before and after nCRT, together with their changes (∆%parameter), between different patient groups (pCR *versus* non-pCR, and GR *versus* PR). Spearman’s rank correlation test was performed to reveal the possible relationship between the TRG score and IVIM-DWI parametric values. The diagnostic performance for the IVIM-DWI parametric values in discriminating the pathologic response to nCRT was assessed using receiver-operating characteristic (ROC) curve analyses. The optimal cut-off value, sensitivity and specificity were determined per the Youden index. All data were analysed using SPSS version 19.0 (SPSS Inc., Chicago, IL) or MedCalc version 15.0 (MedCalc Software bvba, Ostend, Belgium) software. A *P* value < 0.05 was considered statistically significant before multiple comparison correction. The Benjamini-Hochberg false discovery rate (FDR) controlling the procedure was performed with *q* = 0.05 for multiple comparison correction. Therefore, adjusted *P* values < 0.0250 (2*0.05/4), 0.0083 (2*0.05/12), and 0.0083 (2*0.05/12) were regarded as statistically significant for the comparisons of IVIM-DWI parametric values (pre- *versus* post-nCRT, pCR *versus* non-pCR, and GR *versus* PR, respectively)^21^.

## Results

Of the 45 patients who were initially enrolled, 3 were excluded because of a lack of TME operation after nCRT (n = 2) or poor IVIM-DWI imaging quality (n = 1). The present study finally enrolled 42 patients with a mean age of 53 years (range, 26–73 years). The clinical and pathological characteristics of the 42 patients are summarized in Table 1.

**Table 1:** Clinical and pathological characteristics of the enrolled 42 patients.

Variable	Number of Patients
Sex	
Male	34
Female	8
Degree of pathological differentiation	
High differentiation	5
Middle differentiation	29
poorly differentiated	8
Post-nCRT pathologic T (ypT) classification	
ypT0	10
ypT1	0
ypT2	9
ypT3	15
ypT4	8
Post-nCRT pathologic N (ypN) classification	
ypN0	21
ypN1	13
ypN2	8
Pathological response to nCRT	
TRG 4	10
TRG 3	5
TRG 2	16
TRG 1	11
TRG 0	0

nCRT, neoadjuvant chemoradiotherapy; TRG, tumor regression grade.

The intra-class correlation coefficients (95% CI) of inter-observer reproducibility for the measurements of preADC, preD, preD*, pre*f*, postADC, postD, postD* and postf were 0.915 (0.841 to 0.954), 0.912 (0.835 to 0.952), 0.754 (0.542 to 0.868), 0.856 (0.732 to 0.922), 0.872 (0.761 to 0.931), 0.870 (0.759 to 0.930), 0.701 (0.445 to 0.840) and 0.839(0.701 to 0.914), respectively, demonstrating good inter-observer reproducibility and consistency.

For the ultimately included 42 patients, the ADC, D, D* and *f* values of rectal cancers before nCRT were (1.23 ± 0.14) × 10^−3^ mm^2^/s, (0.91 ± 0.11) × 10^−3^ mm^2^/s, (45.24 ± 34.97) × 10^−3^ mm^2^/s and 0.18 ± 0.04, respectively. After nCRT, the ADC, D, D* and *f* values were (1.86 ± 0.36) × 10^−3^ mm^2^/s, (1.43 ± 0.27) × 10^−3^ mm^2^/s, (50.57 ± 35.00) × 10^−3^ mm^2^/s and 0.21 ± 0.10, respectively. Between pre-nCRT and post-nCRT, there were significant differences in the ADC and D values (all *P* < 0.001), whereas no significant differences were found in the D* and *f* values (*P* = 0.464 and 0.069, respectively).

**Figure 1:**
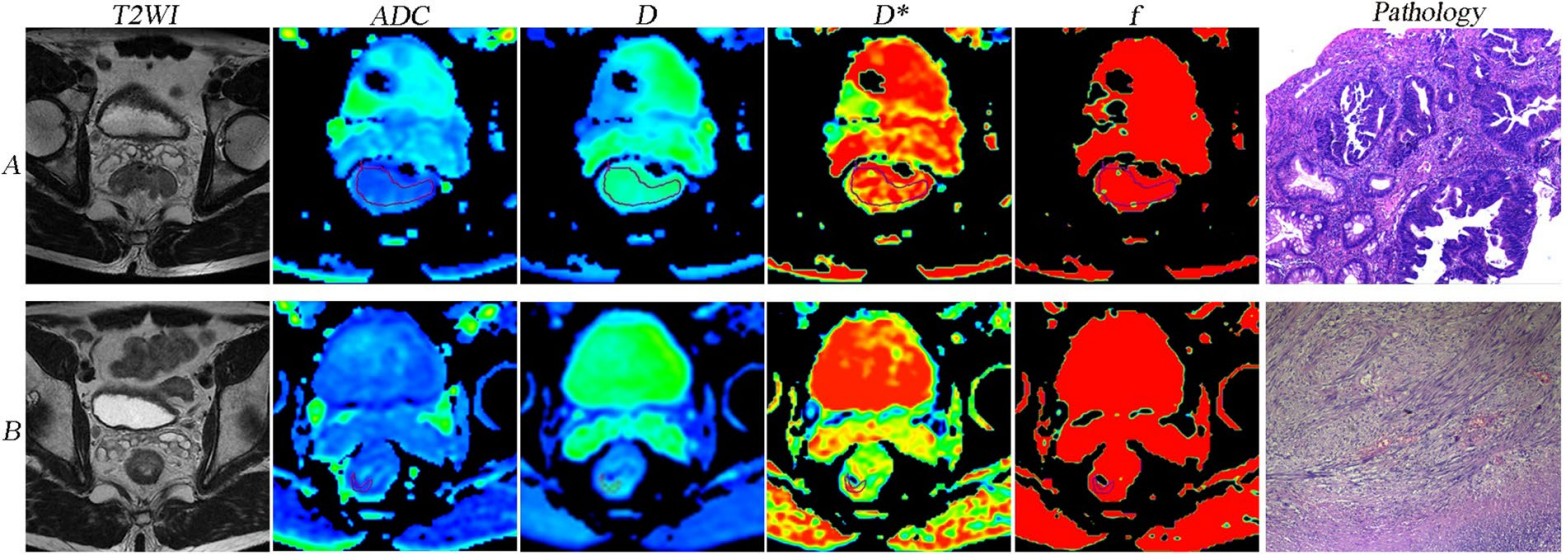
A LARC patient from the pCR and GR groups (TRG 4). Images in the row A and B are T2WI, IVIM-DWI parametric and pathological maps before and after nCRT, respectively. The ADC, D, D* and *f* values before nCRT were 1.07 × 10^−3^ mm^2^/s, 0.785 × 10^−3^ mm^2^/s, 47.7 × 10^−3^ mm^2^/s and 0.206, respectively. The ADC, D, D*, and *f* values after nCRT were 2.28 × 10^−3^ mm^2^/s, 1.81 × 10^−3^ mm^2^/s, 55.5 × 10^−3^ mm^2^/s and 0.248, respectively. The pathological map (haematoxylin-eosin staining, original magnification x40) after nCRT indicates the absence of residual cancer (TRG 4). LARC, locally advanced rectal cancer; pCR, pathological complete response; GR, good response; TRG, tumour regression grade; T2WI, T2-weighted imaging; IVIM-DWI, intravoxel incoherent motion diffusion-weighted imaging; nCRT, neoadjuvant chemoradiotherapy; ADC, apparent diffusion coefficient; D, pure diffusion coefficient; D*, pseudo-diffusion coefficient; *f*, perfusion fraction.

**Figure 2:**
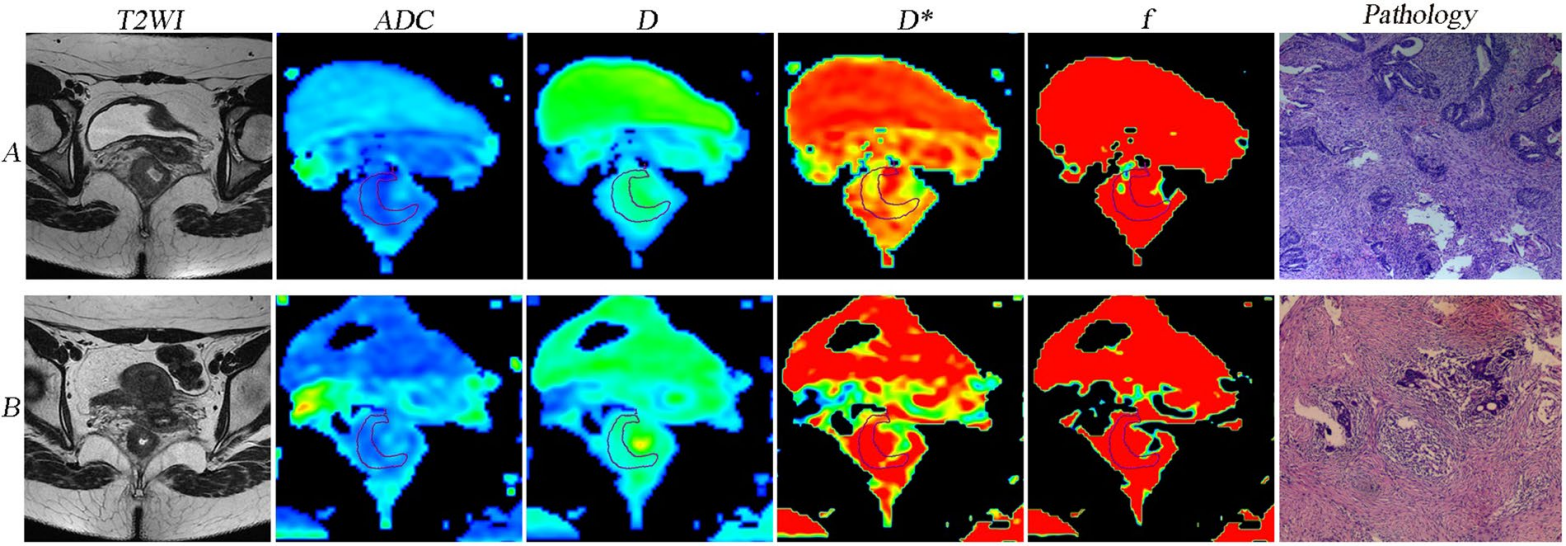
A LARC patient from the non-pCR and PR groups (TRG 2). Images in row A and B are T2WI, IVIM-DWI parametric and pathological maps before and after nCRT, respectively. The ADC, D, D* and *f* values before nCRT were 1.1 × 10^−3^ mm^2^/s, 0.925 × 10^−3^ mm^2^/s, 40.3 × 10^−3^ mm^2^/s and 0.165, respectively. The ADC, D, D*, and *f* values after nCRT were 1.69 × 10^−3^ mm^2^/s, 1.61 × 10^−3^ mm^2^/s, 37.1 × 10^−3^ mm^2^/s and 0.118, respectively. The pathology map (haematoxylin-eosin staining, original magnification x40) after nCRT indicates an increased number of residual cancer cells with predominating fibrosis (TRG 2). LARC, locally advanced rectal cancer; non-pCR, non-pathological complete response; PR, poor response; TRG, tumour regression grade; T2WI, T2-weighted imaging; IVIM-DWI, intravoxel incoherent motion diffusion-weighted imaging; nCRT, neoadjuvant chemoradiotherapy; ADC, apparent diffusion coefficient; D, pure diffusion coefficient; D*, pseudo-diffusion coefficient; *f*, perfusion fraction.

The TRG score revealed a positive correlation with pre*f* (*r* = 0.357, *P* = 0.020), postD (*r* = 0.551, *P* < 0.001) and ∆%D (*r* = 0.605, *P* < 0.001) but no significant correlation with other IVIM-DWI parametric values (Table 2). The IVIM-DWI parametric values for the pCR (n = 10), non-pCR (n = 32), GR (n = 15) and PR (n = 27) groups are summarized in Table 3. The preD*, pre*f*, postD, ∆%ADC and ∆%D values demonstrated significant differences between the pCR and non-pCR groups, whereas the postD, ∆%ADC and ∆%D values showed obvious differences between the GR and PR groups (Table 3). Figures 1 and 2 show representative images of LARC from different pathological response groups.

**Table 2:** Correlation between TRG score and the IVIM-DWI parametric values.

Parameter	*r*	*P*
preADC	−0.090	0.571
preD	−0.005	0.977
preD*	0.172	0.275
pre*f*	0.357	0.020
postADC	0.204	0.194
postD	0.551	<0.001
postD*	0.112	0.481
post*f*	0.043	0.785
∆%ADC	0.252	0.107
∆%D	0.605	<0.001
∆%D*	0.017	0.914
∆%*f*	−0.046	0.774

TRG,tumor regression grade; IVIM-DWI, intravoxel incoherent motion diffusion-weighted imaging; ADC, apparent diffusion coefficient; D, pure diffusion coefficient; D*, pseudo-diffusion coefficient; *f*, perfusion fraction.

**Table 3:** Differences in the IVIM-DWI parametric values between the pCR and non-pCR groups and between the GR and PR groups.

Parameter	pCR (n = 10)	non-pCR (n = 32)	*P*	GR (n = 15)	PR (n = 27)	*P*
preADC (x10^−3^ mm^2^/s)	1.20 ± 0.18	1.25 ± 0.13	0.406	1.21 ± 0.16	1.25 ± 0.13	0.503
preD (×10^−3^ mm^2^/s)	0.88 ± 0.13	0.91 ± 0.11	0.154	0.92 ± 0.15	0.90 ± 0.09	0.937
preD* (×10^−3^ mm^2^/s)	74.64 ± 38.72	47.87 ± 31.71	0.020*	60.54 ± 40.47	50.74 ± 31.79	0.431
pre*f*	0.21 ± 0.04	0.18 ± 0.04	0.045*	0.20 ± 0.04	0.18 ± 0.04	0.059
postADC (×10^−3^ mm^2^/s)	1.94 ± 0.32	1.83 ± 0.37	0.423	1.93 ± 0.29	1.82 ± 0.40	0.282
postD (×10^−3^ mm^2^/s)	1.64 ± 0.25	1.36 ± 0.24	0.004*^#^	1.61 ± 0.26	1.32 ± 0.22	0.002*^#^
postD* (×10^−3^ mm^2^/s)	62.63 ± 42.50	46.79 ± 32.16	0.192	60.72 ± 38.22	44.92 ± 32.45	0.112
post*f*	0.23 ± 0.15	0.21 ± 0.07	0.805	0.22 ± 0.12	0.21 ± 0.07	0.470
∆%ADC (%)	63.19 ± 24.36	47.96 ± 32.79	0.042*	60.34 ± 25.05	46.72 ± 33.89	0.032*
∆%D (%)	88.51 ± 24.54	48.82 ± 22.25	< 0.001*^#^	77.08 ± 27.85	47.83 ± 22.91	0.001*^#^
∆%D* (%)	−4.98 ± 54.85	60.24 ± 228.51	0.738	57.91 ± 187.87	37.38 ± 208.09	0.181

*Significance before multiple comparison correction; ^#^significance after multiple comparison correction (*P*_[2]_ = 0.004 < i*q/m = 2*0.05/12 = 0.0083 for the comparisons between the pCR and non-pCR groups, and *P*_[2]_ = 0.002 < i*q/m = 2*0.05/12 = 0.0083 for the comparisons between the GR and PR groups) according to the Benjamini- Hochberg procedure^21^; IVIM-DWI,intravoxel incoherent motion diffusion-weighted imaging; pCR, pathological complete response; non-pCR, non-pathological complete response; GR, good response; PR, poor response; ADC, apparent diffusion coefficient; D, pure diffusion coefficient; D*, pseudo-diffusion coefficient; *f*, perfusion fraction.

Based on ROC curve analysis, the diagnostic performance of the IVIM-DWI parameters in identifying pathological responses are shown in Tables 4 and 5. To discriminate pCR from non-pCR, ∆%D had the highest area under the curve (AUC) (0.881), sensitivity (90.0%) and positive predictive value (95.9%) among the five IVIM-DWI parameters (preD*, pre*f*, postD, ∆%ADC and ∆%D), which could benefit the identification of pCR to nCRT. The AUC value of ∆%D was significantly higher than that of ∆%ADC in discriminating the pCR from non-pCR groups (*P* = 0.009). Among the three IVIM-DWI parameters (postD, ∆%ADC and ∆%D), which were helpful in distinguishing the good from poor responders. postD had the highest specificity (100%) and positive predictive value (100%) with an AUC of 0.790, whereas ∆%D had the highest sensitivity (93.3%) and negative predictive value (94.1%) with an AUC of 0.807.

**Table 4:** Diagnostic efficacy of the IVIM-DWI parametric values in the differentiation between the pCR and non-pCR groups.

Parameter	AUC (95% CI)	Cut-off value	Sensitivity	Specificity	PPV	NPV	*P*
preD*	0.744(0.574–0.913)	72.20 × 10^−3^ mm^2^/s	60.0%	84.4%	54.5%	87.1%	0.804^a^, 0.674^b^
pre*f*	0.713(0.529–0.896)	0.20	80.0%	71.9%	47.1%	92.0%	0.977^c^, 0.069^d^
postD	0.797(0.626–0.968)	1.67 × 10^−3^ mm^2^/s	60.0%	96.9%	85.7%	88.6%	0.281^e^, 0.273^f^
∆%ADC	0.716(0.556–0.875)	47.6%	80.0%	65.6%	42.1%	91.3%	0.009^g#^, 0.801^h^
∆%D	0.881(0.776–0.986)	65.8%	90.0%	75.0%	52.9%	95.9%	0.125^i^, 0.394^j^

^#^Significance before multiple comparison correction, but not significant after correction; IVIM-DWI, intravoxel incoherent motion diffusion-weighted imaging; pCR, pathological complete response; non-pCR, non-pathological complete response; ADC, apparent diffusion coefficient; D, pure diffusion coefficient; D*, pseudo-diffusion coefficient; *f*, perfusion fraction; AUC, area under the curve; CI, confidence interval; PPV, positive predictive value; NPV, negative predictive value; ^a^preD* vs pre*f*, ^b^preD* vs postD, ^c^pre*f* vs ∆%ADC, ^d^pre*f* vs ∆%D, ^e^pre*f* vs postD, ^f^postD vs ∆%D, ^g^∆%ADC vs ∆%D, ^h^preD* vs ∆%ADC, ^i^preD* vs ∆%D, ^j^postD vs ∆%ADC.

**Table 5:** Diagnostic efficacy of the IVIM-DWI parametric values in the differentiation between the GR and PR groups.

Parameter	AUC (95% CI)	Cut-off value	Sensitivity	Specificity	PPV	NPV	*P*
postD	0.790(0.642–0.939)	1.64 × 10^−3^ mm^2^/s	53.3%	100%	100%	79.4%	0.325^a^
∆%ADC	0.701(0.541–0.861)	47.6%	73.3%	70.4%	57.9%	82.6%	0.113^b^
∆%D	0.807(0.674–0.941)	41.8%	93.3%	59.3%	56.0%	94.1%	0.811^c^

IVIM-DWI, intravoxel incoherent motion diffusion-weighted imaging; GR, good response; PR, poor response; D, pure diffusion coefficient; ADC, apparent diffusion coefficient; AUC, area under the curve; CI, confidence interval; PPV, positive predictive value; NPV, negative predictive value; ^a^postD vs ∆%ADC, ^b^∆%ADC vs ∆%D, ^c^postD vs ∆%D.

## Discussion

This study focused on the feasibility of IVIM-DWI in discriminating the pathological response to nCRT in patients with LARC. Our data found that the pre-nCRT perfusion parametric value (pre*f*) and post-nCRT pure diffusion values (postD and ∆%D) exhibit significant correlations with the pathological response (TRG score) for LARC. Moreover, not only the perfusion-related values at baseline but also the diffusion-related values after nCRT, together with their percentage changes, might benefit the evaluation of the response of LARC to nCRT. Furthermore, one impetus of our study was the finding that IVIM-based D has an advantage over mono-exponential DWI-based ADC in predicting the pathological response of LARC receiving nCRT.

Whether a LARC patient achieves a pCR response to nCRT is an important clinical issue associated with individual treatment. Regarding distinguishing pCR from non-pCR to nCRT for LARC, the present study demonstrated that the baseline diffusion-related parameters (i.e., preADC and preD) may have little potency. Our observations were contrary to previous studies in which rectal cancer with pCR to nCRT exhibited lower baseline ADC values^6, 7^. These contradictory results may suggest the unstable ability of baseline diffusion-related parameters to accurately identify the pathological response of rectal cancer to nCRT.

In the present study, an nCRT-induced evident increase of ADC and D values for all LARC patients was observed. This finding agrees with previous reports on rectal cancer after radiotherapy/chemotherapy^5, 6, 22, 23^. This increase may reflect the cellularity reduction and structural damage of tumour cells as a treatment effect of nCRT^24^. The TRG score can semi-quantitate residual tumour cells after nCRT, and, therefore, directly represents the nCRT-induced anti-tumour effect at the histological level. In this study, the TRG score demonstrated obviously positive correlations with the post-nCRT diffusion-related parametric value (postD and ∆%D), suggesting that the diffusion-related microenvironment in LARC after nCRT has significant relevance to the status of the residual tumour. This suggestion was also supported by our observation that there were obviously higher postD, ∆%ADC and ∆%D values for the pCR (*versus* non-pCR) groups. Prior studies on rectal cancer also revealed a significant addition of postD and/or ∆%ADC values for LARC after nCRT^5–7, 9, 12^. These findings indicate that the pure diffusion parametric values after nCRT may play an important and reliable role in noninvasively identifying a pCR response for LARC preoperatively.

In this study, the pCR group exhibited significantly higher preD* and pre*f* values than the non-pCR group. Additionally, the TRG score revealed a positive correlation with pre*f*. These findings indicate that higher microcirculatory perfusion at baseline might result in better sensitivity to nCRT for LARC. Several previous studies^25–27^ using DCE-MRI also demonstrated that rectal cancer with higher pre-nCRT perfusion will respond favourably to nCRT with a correspondingly longer survival. Higher preD* and pre*f* values represent higher vascularization, perfusion and oxygenation levels in tissues, leading to a better therapeutic response to radiotherapy/chemotherapy. Additionally, higher vascularization and perfusion can result in the better delivery of chemotherapeutics to tumour tissue^28^.

After nCRT, neither the perfusion-related IVIM-DWI parametric values (postD* and post*f*) nor their percentage changes (∆%D* and ∆%*f*) differed significantly between the pCR and non-pCR groups. Moreover, there were no significant correlations between the TRG score and these perfusion-related parametric values. These results suggest that the nCRT-induced change in the IVIM-DWI parametric value involved in perfusion might fail to assess the pathological response for LARC. This failure might result from the complex pattern of nCRT-induced changes in microcirculation perfusion^25, 29, 30^ and poor reproducibility of D* value measurements^31–33^.

Apart from recognizing pCR, the differentiation between GR and PR also has important clinical significance for individualized therapy. A recently published retrospective study investigated the utility of IVIM-DWI in separating GR from PR^23^. Our observations are similar to those of the above study in the post-nCRT perfusion-related parametric values (i.e., post*f*, postD*, ∆%*f* and ∆%D*), but they were different from those before nCRT (i.e., pre*f* and preD*)^23^. Regarding the diffusion-related parameters, our data further confirmed previous findings based on IVIM-DWI or mono-exponential DWI^23, 34–41^; that is, the post-nCRT parametric values (postADC, postD, ∆%ADC and/or ∆%D) rather than baseline values (preADC and/or preD) benefit the differentiation between GR and PR.

Of note, with respect to discriminating tumour pathologic regression defined as pCR *versus* non-pCR, ∆%D had significantly higher AUC values in ROC curve analysis than ∆%ADC. Previous findings demonstrated that ADC based on traditional DWI is less powerful than the IVIM-based parameter D in monitoring the treatment response of numerous malignancies, including nasopharyngeal carcinoma^15^ and breast cancer liver metastases^16^. Additionally, postD can differentiate both pCR (*versus* non-pCR) and GR (*versus* PR) in this study, whereas postADC cannot. Taken together, our study and prior reports might reveal the advantage of D over ADC in predicting the pathological response to nCRT for LARC because D has more time points suitable for this prediction and a higher differentiation performance than ADC.

There are some limitations in our preliminary study. First, our study enrolled a relatively small study population, which probably resulted in statistical bias.Second, the diversity of the TNM stage and differentiation degree was not considered because of the small sample size. It is believed that the treatment response may depend on the TNM stage and degree of differentiation of the tumour. Thus, further studies are needed to perform an analysis of patients with different subgroups stratified by TNM stage and/or degree of differentiation. Third, single-section ROI instead of volumetric evaluation was adopted in the present study. Considering that both analyses produce similar results regarding changes in diffusion measures after CRT and discrimination between good *versus* poor responders to CRT according to a prior report on rectal cancer^23^, we performed the former that is simpler and more practical. However, whole-tumour volume analysis may minimize sampling bias and generate more reproducible IVIM-DWI data than single-section ROI analysis, which may characterize the tumour heterogeneity more accurately^23^. Furthermore, our study investigated the utility of IVIM-DWI only at 2 time points (baseline and 8 weeks after the end of nCRT). Until now, it is unknown which is the best time point for IVIM-DWI in evaluating the treatment response to nCRT in LARC. In future studies, IVIM-DWI should be performed at more time points, such as during and at the end of nCRT, to optimize the IVIM-DWI follow-up scheme.

In conclusion, the present study demonstrates that IVIM-DWI is potentially useful in discriminating the pathological response to nCRT for LARC patients.
